# A novel approach for the router nodes placement in wireless mesh networks using phasing with approximation optimization algorithms

**DOI:** 10.1371/journal.pone.0318247

**Published:** 2025-01-28

**Authors:** Le Huu Binh, Thuy-Van T. Duong, Vuong M. Ngo

**Affiliations:** 1 Faculty of Information Technology, University of Sciences, Hue University, Hue City, Vietnam; 2 Faculty of Information Technology, Ton Duc Thang University, Ho Chi Minh City, Vietnam; 3 Ho Chi Minh City Open University, Ho Chi Minh City, Vietnam; SASTRA Deemed University: Shanmugha Arts Science Technology and Research Academy, INDIA

## Abstract

Optimal router node placement (RNP) is an effective method for improving the performance of wireless mesh networks (WMN). However, solving the RNP problem in WMN is difficult because it is NP-hard. As a result, this problem can only be solved using approximate optimization algorithms such as heuristics and meta-heuristics. In this study, we propose a new and effective method for solving the RNP problem. The idea behind this method is to solve the RNP problem in two stages using an optimal algorithm with fewer variables than the original RNP problem. In stage 1, we build an RNP sub problem using 15% to 20% of the number of routers, with the objective function of minimizing coverage overlap between routers to form a core network. Stage 2 is built into another RNP sub problem with the remaining number of routers, and the objective function is to maximize the network connectivity. Each sub problem was solved using an approximate optimal algorithm. The experimental results demonstrate that, in terms of client coverage and network connectivity, our proposed method outperforms widely used RNP problem-solving methods.

## Introduction

**Fig 1:**
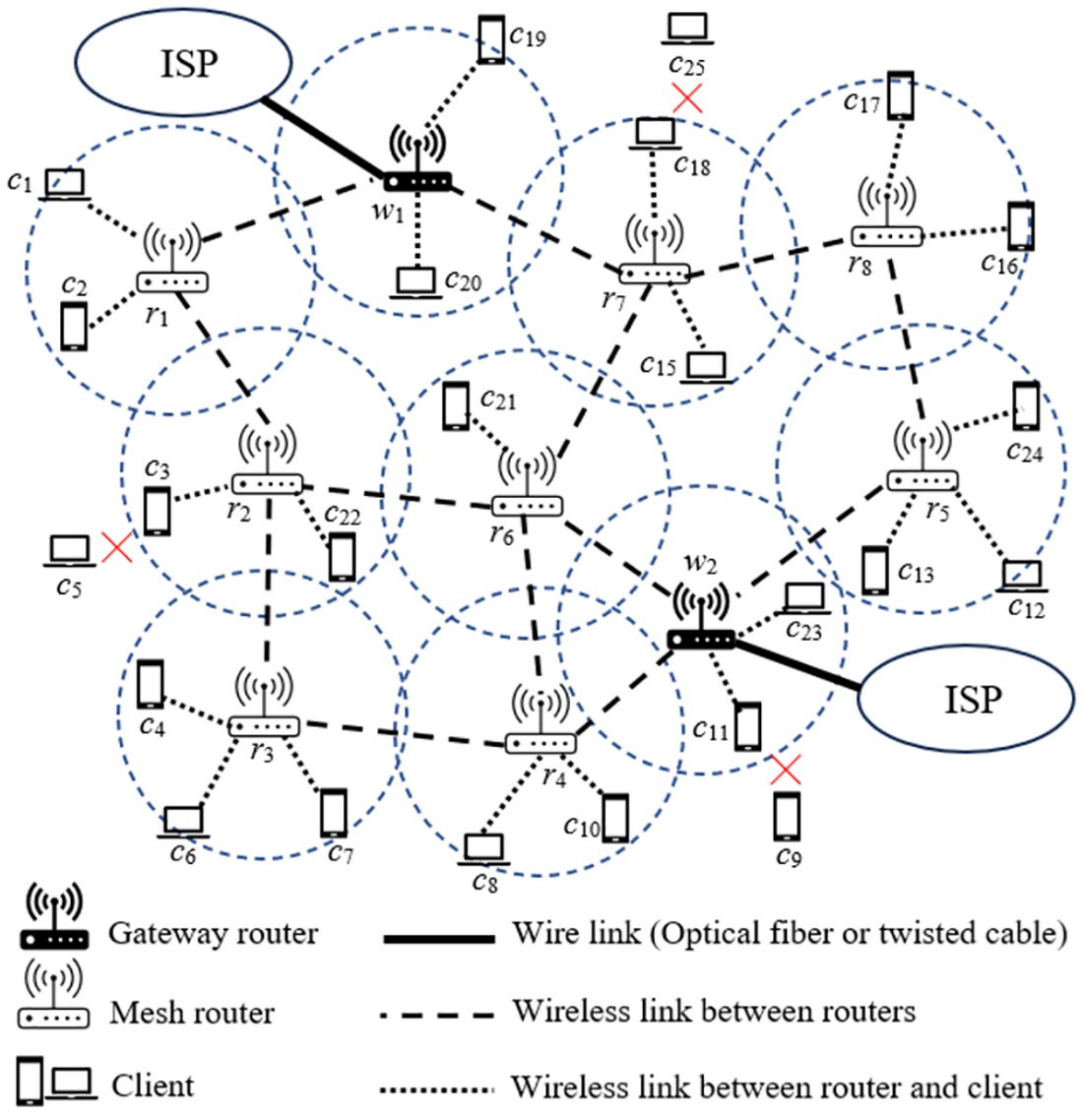
A wireless mesh network consists of two gateway routers, eight mesh routers and twenty-one clients.

Wireless mesh networks (WMN) are currently the main technology solution in wireless local area networks (WLAN) of agencies, businesses, and schools. WMN is being prioritized by network administrators because it has many advantages compared to wireless networks using traditional access points, typically reducing congestion due to the ability to balance load, and convenience in deploying infrastructure because there is no need to connect wired links to all wireless routers. Fig 1 shows an example of a WMN consisting of two gateway mesh router (GMR) (*w*_1_ and *w*_2_), eight mesh wireless routers (*r*_1_ to *r*_8_), and twenty-one clients. Two GWRs, *w*_1_ and *w*_2_, are connected directly to the Internet service provider (ISP) via an optical fiber or a twisted pair cable. In a WMN, the number of GMRs is usually determined based on the average traffic load in the network and the capacity of the connection channels from the GMRs to the ISP, as shown in Fig 1, which are channels from *w*_1_ and *w*_2_. The remaining wireless routers (*r*_1_ to *r*_8_) connect to each other through wireless links, forming a mesh topology. Each client connects to a wireless router to access Internet. If a client is covered by multiple routers, it connects to the router with the strongest signal strength.

Designing and deploying a WMN network with the best performance is essential to satisfy the current demand for wireless network services. This has motivated many research groups to focus on WMN recently. Network topology control [1–4], router node placement (RNP) [5–20], routing protocols [21–24], and access point allocation [25–28] are some of the most common topics that have been deployed, in which the RNP problem is the most interesting topic. As this problem is NP-hard [10], conventional algorithms cannot solve it. The use of approximate optimization algorithms, such as heuristics and meta-heuristics is one possible solution to this problem. Several approximation algorithms have been successfully applied to solve the RNP problem, such as the Multi-Verse Optimizer algorithm (MVO) [8], particle swarm optimizer algorithm (PSO) [6], accelerated particle swarm optimizer algorithm (APSO) [6], linearly decreasing weight particle swarm optimizer (LDWPSO) [29], the coyote optimization algorithm (COA) [5], Chemical Reaction Optimization algorithm (CRO) [7]. These studies have solved the RNP problem, and the results obtained can be applied to install WMN in practice. However, in the case of WMN with a large area, many mesh routers, and dense client density, solving the RNP problem using approximate algorithms is often difficult because of the increase in the number of variables and search space, leading to increased computational complexity. Therefore, it is necessary to investigate how to effectively apply approximation optimization algorithms to the RNP problem in these cases. This motivated us to conduct this study. The main contributions of this study are summarized as follows.

(i) We propose a new objective function for the RNP problem that contains two metrics, NC and COR.
(ii) We propose a new and effective method to solve the RNP problem. Our idea is to solve the RNP problem in two stages using fewer variables than the original RNP problem. In stage 1, we build an RNP sub problem using 15% to 20% of the number of routers, with the objective function of minimizing COR to form a core network. Stage 2 is built into another RNP sub problem with the remaining number of routers, and the objective function is to maximize NC. Each sub-problem is solved using an approximate optimal algorithm.

The remainder of this paper is organized as follows. The next section presents the RNP problem. The following sections describes the proposed method to solve the RNP problem and experimental results. The final section is conclusions and suggestions for further research.

## Related works

Recently, a lot of researchers have been interested in finding a solution to the RNP problem in WMN. Numerous methods have been put up to address this issue, most commonly including the use of reinforcement learning and approximate optimization algorithms. Using reinforcement learning, the authors of [15] proposed a highly effective method to solve the RNP problem. The authors modeled the RNP problem as a reinforcement learning process. The features for reinforcement learning are {environment, agent, action, and reward}, which correspond to {network system, mesh routers, coordinate adjustment of mesh routers, and network connectivity} of the RNP problem. Simulation results indicate that as compared to current methods, the suggested method has greatly enhanced network connection. Approximate optimization algorithms are another method that researchers have recently employed to solve the RNP problem. A recent study by Sylia M. T. et al. used the COA to address the RNP problem [5]. In this study, an objective function made up of two performance metrics, network connectivity and user convergence, is developed. The RNP problem is then reformulated as a nonlinear programming problem with the purpose of maximizing the two specified metrics. The outcomes of the simulation show that, in comparison to alternative optimization methods, COA is more beneficial and successful at determining the best coordinates for mesh routers. Another study recommended using the MVO algorithm to solve the RNP problem [8]. The authors of this study suggested a new objective function for the RNP issue that takes into account the connected client ratio and connected router ratio metrics, two crucial performance indicators. Next, in order to maximize the two suggested metrics, the mesh routers are placed using a coordinate set that is determined by the MVO method. The findings of the experiment indicate that, in comparison to the other optimization algorithm, the MVO algorithm improves the connected client ratio and decreases the path loss of the wireless links when it is used to the RNP problem. In [30], the authors suggested an improved Moth Flame Optimization (MFO) algorithm for solving the RNP issue. This technique is known as Enhanced Chaotic Lévy Opposition-based MFO (ECLO-MFO). Taking into account network connectivity and client coverage metrics, the suggested ECLO-MFO’s efficacy is evaluated in a variety of scenarios and configurations. In comparison with the original MFO and 10 other optimization algorithms, the simulation results show the accuracy and superiority of ECLO-MFO in selecting the ideal placements of mesh routers. Also with the method of applying approximate optimization algorithm to RNP problem, an intelligent simulation system based on the Cuckoo Search (CS) algorithm, WMN-CS, was proposed and put into use by the authors of [31]. By adjusting the scale parameter (*γ*) and host bird discovery rate (*p*_*a*_), this study assessed WMN-CS performance for different combinations of hyperparameters. The simulation findings indicate that when the host bird recognition rate (*p*_*a*_) is between 0.900 and 0.925 and the scale parameter (*γ*) is between 0.07 and 0.09, greater performance is obtained.

The findings of the aforementioned studies suggest that using approximate optimization methods to address the RNP problem is a feasible and highly successful strategy. However, in the case of WMN with a vast area, multiple mesh routers, and dense client density, solving the RNP problem with approximate methods is frequently challenging due to a rise in the number of variables and search space, resulting in increased computing complexity. As a result, It is essential to look into the best ways to solve the RNP problem using approximation optimization algorithms in these instances. This problem is addressed in this study by a novel approach to solving the RNP problem which is implemented in two stages. The following sections present the proposed solution in detail.

## RNP problem

### Graph theoretical model for WMN

Consider a WMN consisting of *m* mesh routers, *n* clients, and *k* gateway routers located in an area of *H* × *W* m. We used an undirected graph *G*(*V*, *E*) to represent this WMN, where *V* and *E* denote the vertex and edge sets, respectively. *V* = *R* ∪ *W* ∪ *C*, where *R* = {*r*_*i*_|*i* = 1..*m*} is the set of mesh routers, *W* = {*w*_*i*_|*i* = 1..*k*} is the set of gateway routers and *C* = {*c*_*i*_|*i* = 1..*n*} denotes the set of clients. *E* = *L*_*r*,*r*_ ∪ *L*_*r*,*w*_ ∪ *L*_*r*,*c*_, where *L*_*r*,*r*_ is the set of wireless links between mesh routers, *L*_*r*,*w*_ is the set of wireless links between mesh routers and gateway routers, and *L*_*r*,*c*_ is the set of wireless links between mesh routers and clients.

For the RNP problem, the network connectivity metric is often used to evaluate network performance [5, 6, 8]. This metric was also used in this study. In addition, we used an additional metric of overage overlap between routers for the core network design stage.

### Network connectivity

In a WMN, network connectivity is defined as the ratio of the number of connected clients to the number of clients in the network. Let *NC* be the network connectivity; then, *NC* is determined by

$$\begin{array}{c} NC=\frac{|C_{c}|}{\left|C\right|}\times 100\% \end{array}$$ (1)

where *C*_*c*_ denotes the set of clients connected to at least one gateway router. Returning to the example of WMN in Fig 1, sets *C* and *C*_*c*_ are determined as follows

$$\begin{array}{cc} & C=\{c_{i}|i=\bar{1..25}\} \end{array}$$ (2)

$$\begin{array}{cc} & C_{c}=\left\{C\backslash \left\{c_{5},c_{9},c_{25}\right\}\right\} \end{array}$$ (3)

because *c*_5_, *c*_9_ and *c*_25_ are not connected to any gateway router. According to (1) we have:

$$\begin{array}{c} NC=\frac{|C_{c}|}{\left|C\right|}\times 100\%=\frac{22}{25}\times 100\%=88\% \end{array}$$ (4)

### CoOverage overlap radius between mesh routers

**Fig 2:**
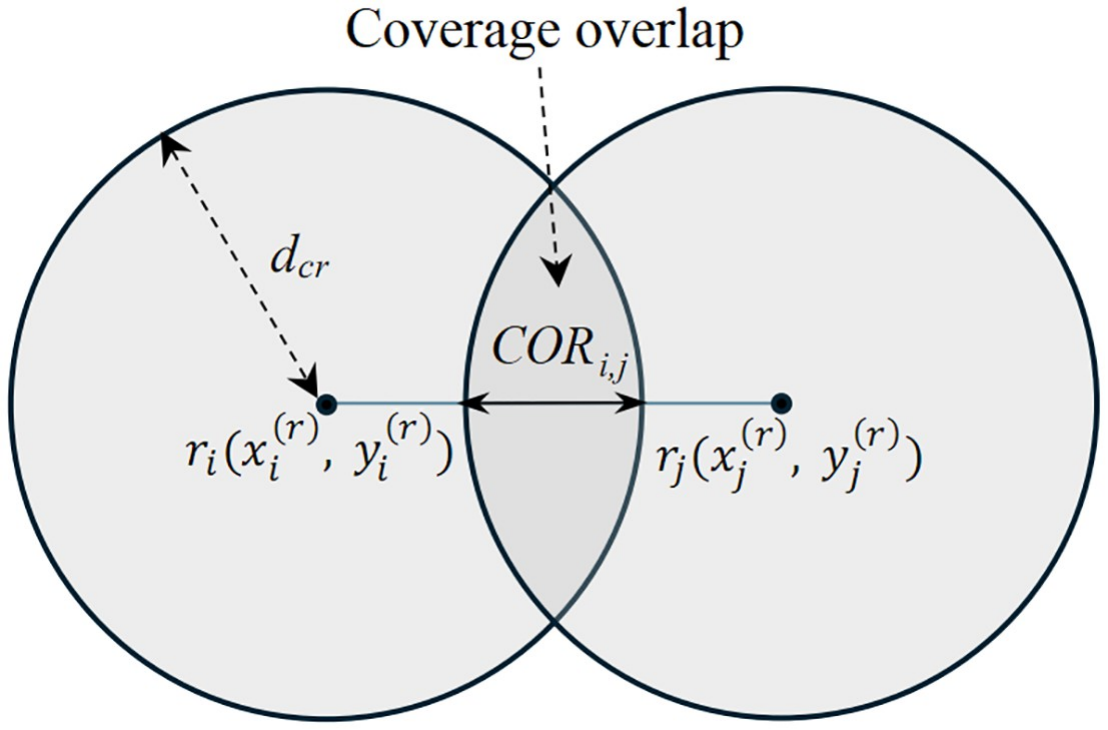
Illustration of coverage overlap between two mesh routers.

In a WMN, the coverage overlap radius (COR) between routers significantly affects the coverage area of the entire network. Fig 2 illustrates the COR between the two mesh routers *r*_*i*_ and *r*_*j*_, calculated as

$$\begin{array}{c} COR_{i,j}=\{\begin{array}{cc} 2d_{cr}-d_{i,j} & if\;d_{i,j}<2d_{cr}\\ 0 & otherwise \end{array} \end{array}$$ (5)

where *d*_*cr*_ is the coverage radius of each mesh router, and *d*_*i*,*j*_ is the distance between mesh routers *r*_*i*_ and *r*_*j*_. determined by

$$\begin{array}{c} d_{i,j}=\sqrt{{\left(x_{i}^{\left(r\right)}-x_{j}^{\left(r\right)}\right)}^{2}+{\left(y_{i}^{\left(r\right)}-y_{j}^{\left(r\right)}\right)}^{2}} \end{array}$$ (6)

where $\left(x_{i}^{\left(r\right)},y_{i}^{\left(r\right)}\right)$ and $\left(x_{j}^{\left(r\right)},y_{j}^{\left(r\right)}\right)$ are the coordinates of the mesh routers *r*_*i*_ and *r*_*j*_, respectively.

### Formulation of the RNP problem

In this section, we formulate the RNP as a nonlinear programming problem. Consider the WMN described in the perivous section. Given $C_{c}=\left\{\left(x_{i}^{\left(c\right)},y_{i}^{\left(c\right)}\right)|i=1..n\right\}$ and $C_{w}=\left\{\left(x_{i}^{\left(w\right)},y_{i}^{\left(w\right)}\right)|i=1..k\right\}$ are the sets of coordinates of *n* clients and *k* gateway routers. The RNP problem is stated as finding a set $C_{r}=\left\{\left(x_{i}^{\left(r\right)},y_{i}^{\left(r\right)}\right)|i=1..m\right\}$ which is a set of coordinates for placing *m* mesh routers to achieve the specific objectives. In our context, the objectives of the RNP problem are to maximize NC and minimize COR. Consequently, The RNP problem is formulated as a nonlinear programming problem as follows:

$$\begin{array}{c} \left\{ \begin{array}{c} Maximize\;NC\\ Minimize\;(\underset{\forall r_{i},r_{j}\in R}{Max\;}COR_{i,j}) \end{array}\right. \end{array}$$ (7)

subject to

$$\begin{array}{c} lb_{x}\leq x_{r_{i}}\leq ub_{x},\;\left(i=1..m\right) \end{array}$$ (8)

$$\begin{array}{c} lb_{y}\leq y_{r_{i}}\leq ub_{y},\;\left(i=1..m\right) \end{array}$$ (9)

where *lb*_*x*_, *lb*_*y*_ and *ub*_*x*_, *ub*_*y*_ are the lower and upper bounds of search space $x_{r_{i}}$ and $y_{r_{i}}$.

**Fig 3:**
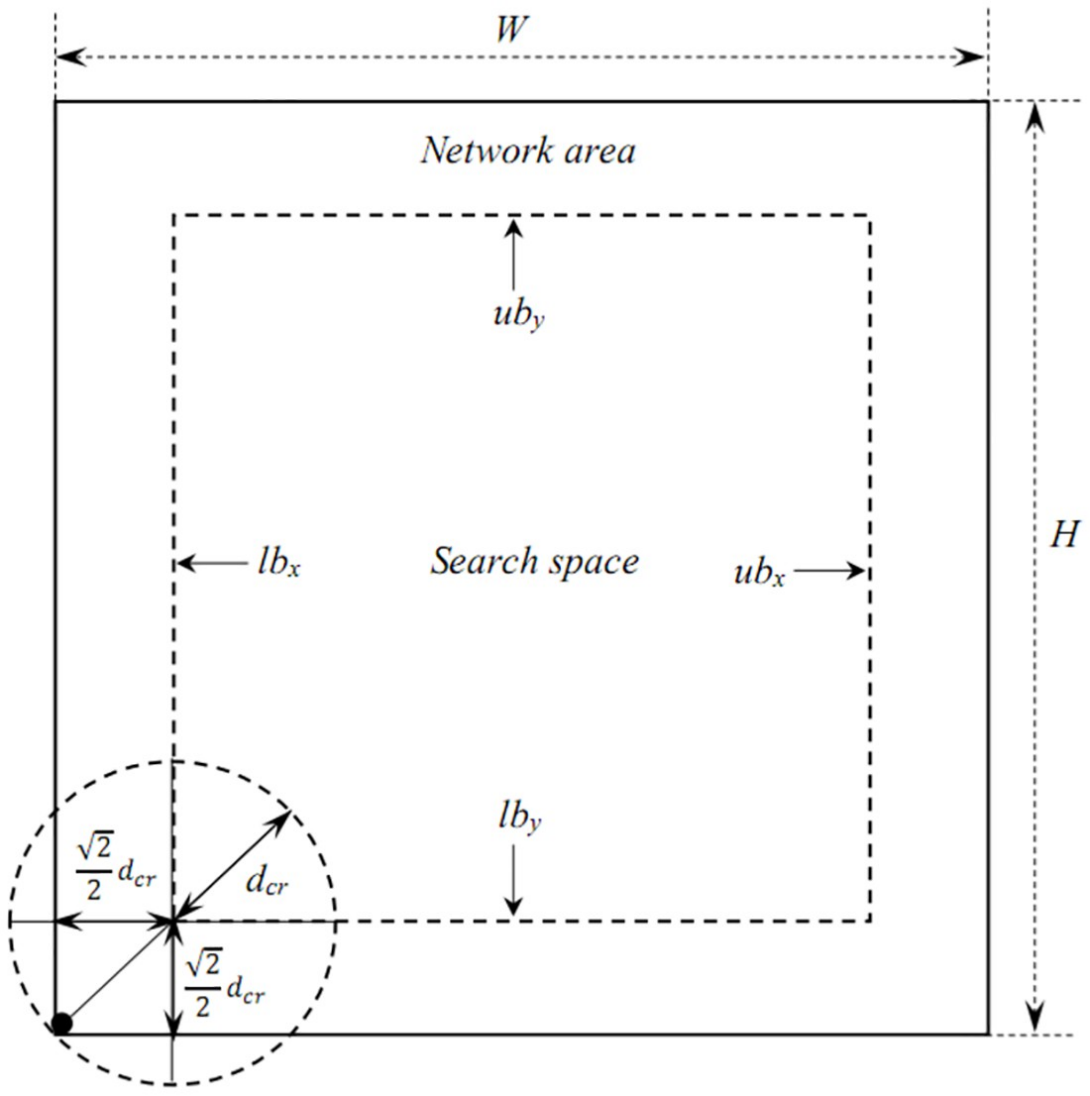
Illustrate the lower and upper bounds of the search space in the RNP problem.

Most previous studies have chosen at search space for the entire network area. This option is reasonable for determining the coordinate solution for mesh routers. However, this is not optimal because in some cases, the router positions are close to the boundaries. In this case, approximately half of the mesh routers’ coverage area extends beyond the network area. This squanders the network resources. In this study, the search space is shrunk enough to cover clients that are close to the boundaries. This reduces the computational complexity and optimizes network resource usage. To determine the optimal lower and upper bounds for the search space, we consider the case in which the client is farthest from the center, that is, at the origin of the network space, as shown in Fig 3. In this case, the router position shown in Fig 3 is sufficient to cover the client, whereas coverage beyond the network area is minimal. The lower and upper bounds of the search space $x_{r_{i}}$ and $y_{r_{i}}$ are determined as follows:

$$\begin{array}{c} lb_{x}=lb_{y}=\frac{\sqrt{2}}{2}d_{cr} \end{array}$$ (10)

$$\begin{array}{c} ub_{x}=W-\frac{\sqrt{2}}{2}d_{cr} \end{array}$$ (11)

$$\begin{array}{c} ub_{y}=H-\frac{\sqrt{2}}{2}d_{cr} \end{array}$$ (12)

where *d*_*cr*_ is the coverage radius of the routers and *W* and *H* are the width and height of the network area, respectively.

## Proposed method

In this section, we present a new method to solve the RNP problem. Our idea was to solve the RNP problem in two stages using an approximate optimization algorithm with fewer variables than the original RNP problem. The first stage places 15% to 20% of the mesh routers in the network area to form a core network with the widest coverage area. Therefore, the objective function of this stage focuses on minimizing COR. Mesh routers that have been successfully placed at this stage act as gateways for the remaining routers that are placed at the next stage. The objective function focuses on maximizing the NC used for the second stage to place all remaining routers in the network area.

### Objective function

As discussed in Section 1, the objective of the NRP problem in our context is to optimize two metrics: *NC* and *COR*. Because most approximate optimization algorithms are designed to solve single-objective minimization problems, the objective function is constructed as follows:

$$\begin{array}{c} f\left(P\right)=\lambda \left(1-NC\right)+\left(1-\lambda \right)\;\underset{\forall r_{i},r_{j}\in R}{Max\;}COR_{i,j} \end{array}$$ (13)

where *λ* is a coefficient in the range [0, 1], that is used to control the optimal degree of the metrics.

**Algorithm 1** Solving RNP problem by Combining Approximate Algorithms and Network Classification

**Input:**

 • Set of clients: *C* = {*c*_*i*_|*i* = 1..*n*} and their coordinates: $P_{c}=\left\{\left(x_{i}^{\left(c\right)},y_{i}^{\left(c\right)}\right)|i=1..n\right\}$;

 • Set of gateway routers: *W* = {*w*_*i*_|*i* = 1..*k*} and their coordinates: $P_{w}=\left\{\left(x_{i}^{\left(w\right)},y_{i}^{\left(w\right)}\right)|i=1..k\right\}$;

 • Set of mesh routers: *R* = {*r*_*i*_|*i* = 1..*m*};

 • Size of network area: *W* × *H*;

**Output**: The best coordinates to place *m* mesh routers: $P_{r}=\left\{\left(x_{i}^{\left(r\right)},y_{i}^{\left(r\right)}\right)|i=1..m\right\}$;

**Method:**

 // *Stage 1*

1: Select $m_{s_{1}}$ mesh routers for set $R_{s_{1}}$ from set *R*;

2: *λ* ← 0.1;

3: $G_{s_{1}}\leftarrow G$;

4: Use an approximate optimization algorithm to solve the RNP problem with input: *C*, $R_{s_{1}}$, $G_{s_{1}}$, *d*_*cr*_, *W*, *H*. Output: $P_{s_{1}}$;

 // *Stage 2*

5: **for** (*each mesh router* $r_{i_{1}}\in R_{s}$) **do**

6:  **if** (*r*_*i*_ *is not connected to any gateway router*) **then**

7:   $R_{s_{1}}\leftarrow R_{s_{1}}\backslash \left\{r_{i}\right\}$;

8:   $P_{s_{1}}\leftarrow P_{s_{1}}\backslash \left\{\left(x_{r_{i}},y_{r_{i}}\right)\right\}$;

9:  **end if**

10: **end for**

11: *λ* ← 0.9;

12: $G_{s_{2}}\leftarrow G\cup R_{s_{1}}$;

13: Determine which clients are already covered by routers in set $R_{s_{1}}$, denoted by set *C*_*covered*_;

14: *C* ← *C*∖{*C*_*covered*_};

15: Use an approximate optimization algorithm to solve the RNP problem with input: *C*, $R_{s_{2}}$, $G_{s_{2}}$, *d*_*cr*_, *W*, *H*. Output: $P_{s_{2}}$;

16: $P_{r}\leftarrow P_{s_{1}}\cup P_{s_{2}}$;

### Proposed algorithm

Algorithm 1 is the pseudo-code of the algorithm used to solve the RNP problem by combining approximate optimization algorithms and network classification. In the first stage, *λ* coefficient in objective function (13) is set to 0.1 to focus on minimizing *COR* metric. The goal of this stage is to find $m_{s_{1}}$ coordinates to place $m_{s_{1}}$ mesh routers to form a core network with the largest coverage area. The mesh routers placed in this stage act as gateways for the mesh routers placed in the second stage. In the second stage, *λ* coefficient in the objective function (13) is set to 0.9 to focus on optimizing the *NC* metric. This allows finding $m_{s_{2}}$ coordinates to place all remaining mesh routers so that the number of clients covered is maximized.

## Performance evaluation

### Simulation scenarios

We implemented a simulation using Python programming language to evaluate the performance of the proposed method. All experiments were carried out on a 3.3 GHz Core i7 CPU machine. Our method is compared with the well-known RNP problem-solving method that employs approximate optimization algorithms, WOA [32] and MVO [33]. All methods are implemented in Python. The simulation assumptions are presented in Table 1. Simulation scenarios are performed in a network area from 2000 × 2000 [*m*^2^] to 2500 × 2500 [*m*^2^]. The number of mesh routers varies from 20 to 45 with a step of 2, and the number of mesh clients varies from 100 to 400 with a step of 25 The parameters of the approximate optimization algorithms are presented in Table 2. These parameters are set as in [8] to provide a basis for comparing simulation results. Because of the randomness of the approximate optimization algorithms, to ensure the accuracy of the simulation results, we execute each scenario 50 times. The results presented in this section are averages of 50 runs.

**Table 1:** The parameters of simulation scenarios.

Parameter	Setting
*λ* in the objective function (13)	0.1 and 0.9 for stage 1 and stage 2, respectively
Number of iteration	1000
Network area	2000 × 2000 [*m*^2^], 2500 × 2500 [*m*^2^]
Number of mesh routers	20:2:45
Number of mesh clients	100:25:400
Coverage radius	200 *m*
Number of runs of each scenario	50

**Table 2:** The parameters of algorithms.

Algorithm	Parameter	Setting
MVO	Universes number	50
	*WEP*	Increase from 0.2 to 1
	*TDR*	Decrease from 0.6 to 0
WOA	Search-agent Number	50
	Control parameter *a*_*max*_	2
	Control parameter *a*_*min*_	0

### Simulation results

**Fig 4:**
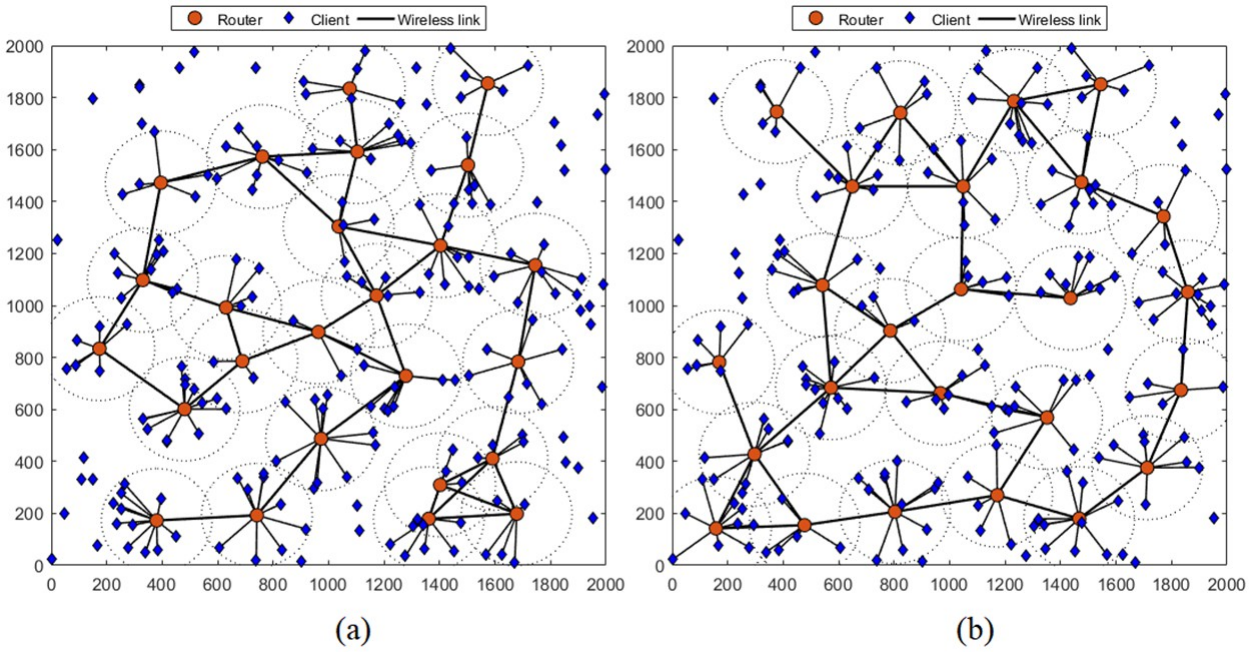
Compare topologies when using the MVO algorithm to solve the RNP problem by different methods. (a) traditional method (one-stage) and (b) proposed method (two-stages).

**Fig 5:**
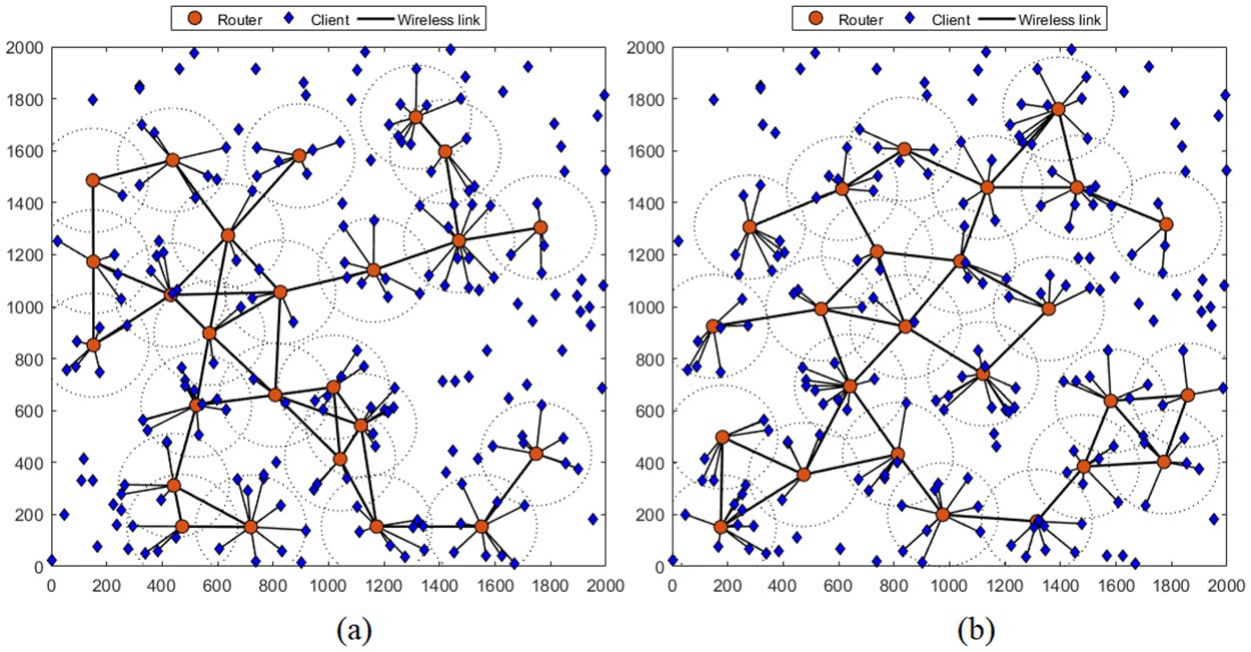
Compare topologies when using the WOA algorithm to solve the RNP problem by different methods. (a) traditional method (one-stage) and (b) proposed method (two-stages).

First, we compare the network topologies obtained using the proposed method versus the traditional method. The results shown in Figs 4 and 5, are performed for the simulation scenario where the number of mesh routers is 25, covering 200 clients in an network area of 2000 × 2000 *m*^2^. We easily observe that the proposed method yields a more optimal topology than the traditional method for both cases where the MVO and WOA algorithms are used. Specifically, considering the case of the MVO algorithm (Fig 4), when using the traditional method, the topology obtained in Fig 4(a), 164 clients are covered, corresponding to a rate of 84%. If the proposed method is used, the number of clients covered is 177, corresponding to a rate of 89.78% (Fig 4(b)). The results are also completely similar for the case of using the WOA algorithm, as shown in Fig 5. The proportion of clients covered is 73.33% and 80.00% for the traditional method and the proposed method, respectively.

**Fig 6:**
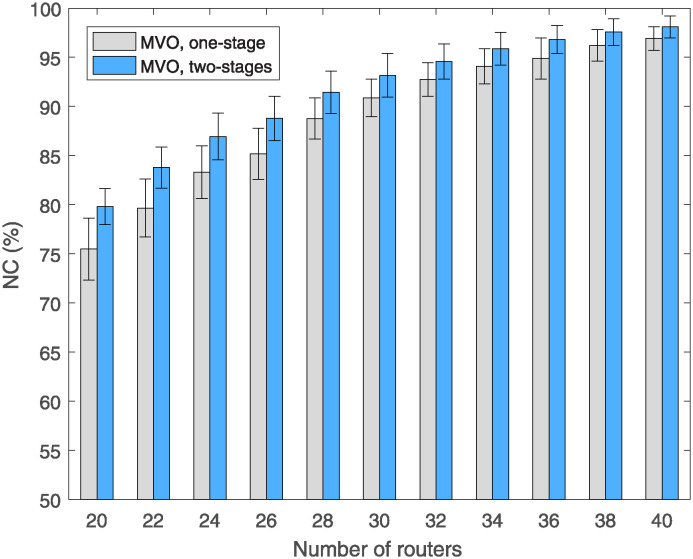
Compare NC versus the number of routers using MVO algorithm with different methods.

Next, we analyze an important metric that is often used to evaluate the effectiveness of the RNP problem solving method, NC, defined as (4). Fig 6 shows the variation of NC versus the number of mesh routers in the case where the number of clients is 150 and the MVO algorithm is used. In this figure, the legends labeled one-stage and two-stages represent the traditional and proposed methods, respectively. We can observe that with both methods, NC increases proportionally to the number of mesh routers, where the proposed method, two-stages, always yields a higher NC than that of the traditional method, one-stage. Considering of 20 mesh routers, the average NC values of the one-stage and two-stages methods are 75.84% and 79.81%, respectively. Thus, the two-stage method improved the NC by 3.97% compared to the one-stage method. When the number of mesh routers increase from 20 to 40, NC of both methods increases, in which the NC of the two-stages method is higher than that of the one-stage method with an average value of 3% to 5%. The specific values are shown in Table 3, where we summarize the statistical data of all 50 replicate runs for each simulation scenario. The increase of NC using the proposed method is a very significant result in improving the WMN performance.

**Table 3:** NC statistics versus total routers over 50 runs replicate per scenario using the MVO algorithm with various approaches.

Nunber of routers	Average NC %		Max NC (%)		Min NC (%)		STD	
	one-stage	two-stages	one-stage	two-stages	one-stage	two-stages	one-stage	two-stages
20	75.48	79.81	81.76	83.53	64.12	75.29	3.14	1.84
22	79.65	83.78	84.88	87.79	72.09	79.07	2.95	2.08
24	83.31	86.94	89.08	91.38	77.59	81.03	2.67	2.40
26	85.18	88.78	90.34	93.75	78.98	84.09	2.60	2.25
28	88.78	91.44	93.26	95.51	83.71	87.64	2.08	2.14
30	90.87	93.17	94.44	97.78	86.11	87.22	1.90	2.20
32	92.75	94.58	97.80	97.80	89.56	90.11	1.71	1.79
34	94.08	95.86	98.37	98.91	89.67	91.85	1.78	1.67
36	94.89	96.82	97.85	98.92	88.71	91.94	2.10	1.43
38	96.21	97.56	99.47	100.00	90.96	94.15	1.59	1.36
40	96.92	98.09	100.00	100.00	94.74	95.26	1.19	1.13

**Fig 7:**
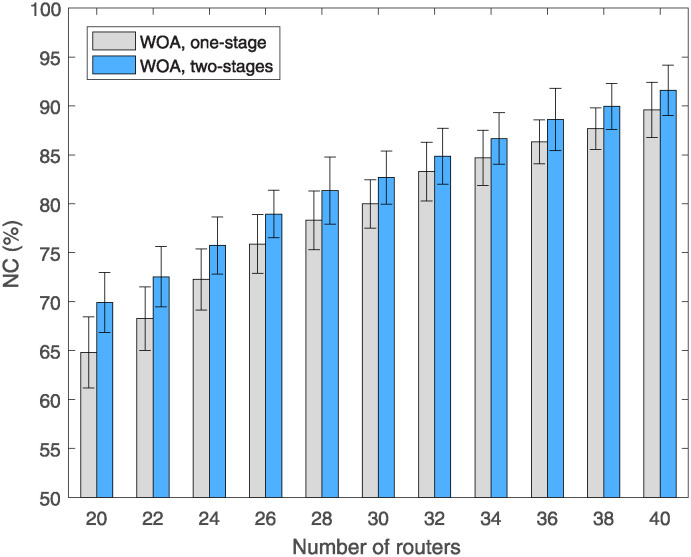
Compare NC versus the number of routers using WOA algorithm with different methods.

In the case of using the WOA algorithm, the results are found as shown in Fig 7. Although NC is not achieved as in the case of the MOV algorithm, if comparing between two methods, the two-stages method always yields higher NC than the one-phase method when the number of mesh routers increases from 20 to 40. The specific data are shown in Table 4, where we compare both the mean, maximum, minimum and standard deviation of 50 replicate runs for each simulation scenario. For the average NC, the proposed method, two-stages, increases NC by 3.1% to 5.1%. compared to the traditional method, one-stage. The simulation scenario with 20 routers had the highest NC increase, at 12%. In this instance, the NCs of the traditional and new methods are 64.82% and 69.91%, respectively. For the remaining simulation scenarios, the average NC also increased significantly.

**Table 4:** NC statistics versus total routers over 50 replicate runs per scenario using the WOA algorithm with various approaches.

Nunber of routers	Average NC %		Max NC (%)		Min NC (%)		STD	
	one-stage	two-stages	one-stage	two-stages	one-stage	two-stages	one-stage	two-stages
20	64.82	69.91	71.76	77.06	52.94	62.94	3.64	3.05
22	68.28	72.55	75.58	80.23	61.05	64.53	3.24	3.08
24	72.26	75.74	79.31	81.03	67.24	70.11	3.12	2.92
26	75.89	78.94	82.39	84.66	69.32	75.00	2.99	2.43
28	78.31	81.34	83.15	88.20	71.91	73.03	3.00	3.42
30	79.99	82.68	87.22	88.33	75.00	77.22	2.47	2.70
32	83.30	84.85	89.01	90.66	76.37	78.57	3.00	2.86
34	84.70	86.67	90.76	91.85	77.72	80.43	2.83	2.63
36	86.32	88.61	90.86	96.24	80.65	82.26	2.24	3.17
38	87.66	89.95	93.62	95.21	82.98	84.04	2.12	2.36
40	89.59	91.60	95.79	96.84	83.68	85.79	2.81	2.57

**Fig 8:**
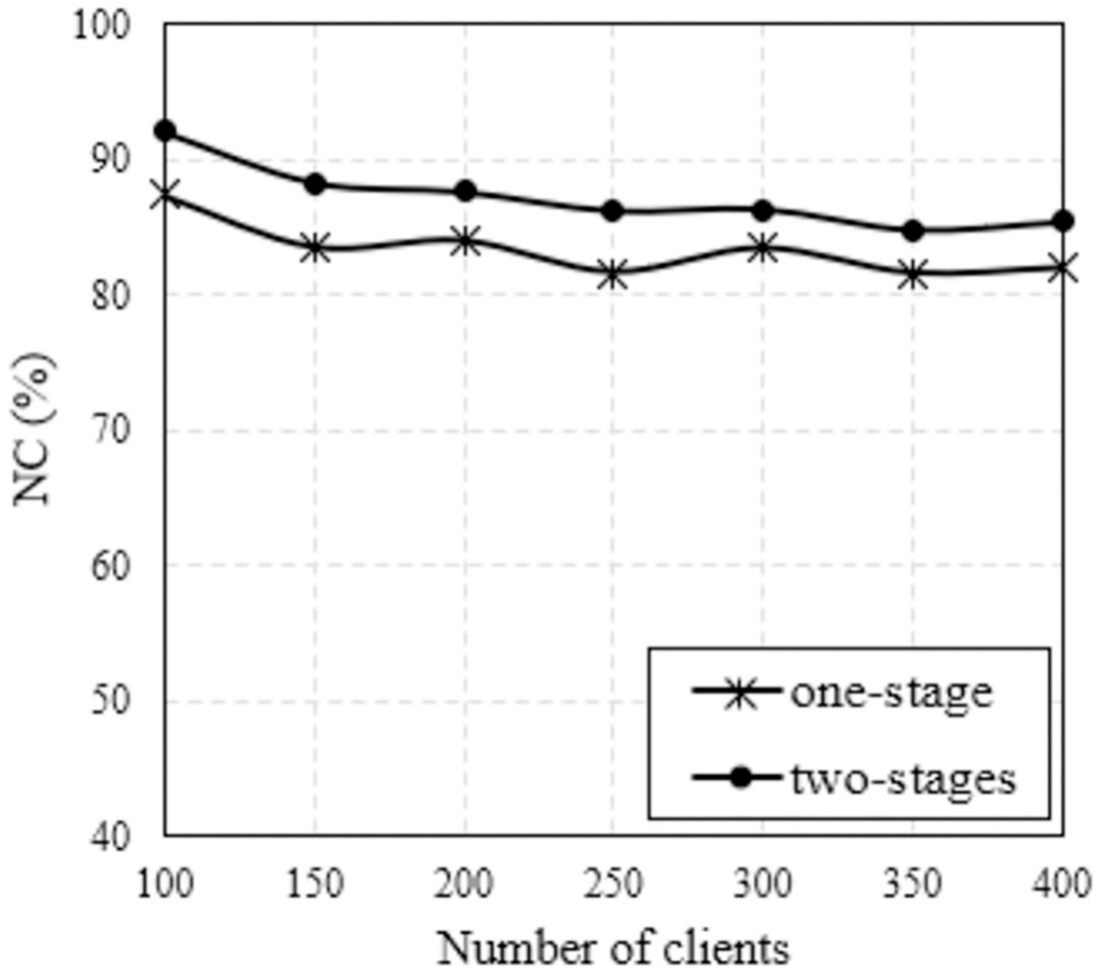
Compare NC versus the number of clients using MVO algorithm with different methods.

**Fig 9:**
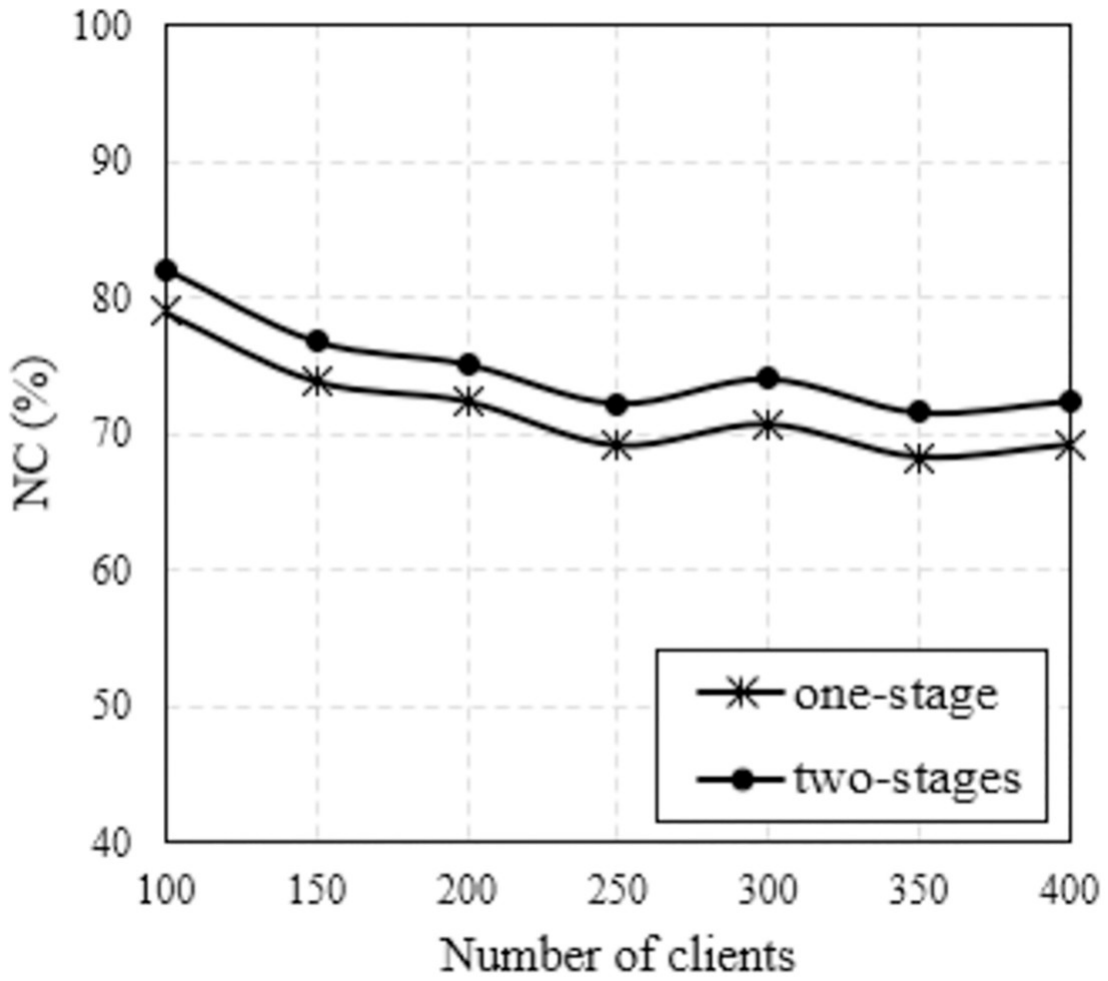
Compare NC versus the number of clients using WOA algorithm with different methods.

Another parameter that affects NC is the number of clients. We carefully investigated these scenarios with the number of clients varies from 100 to 400. As analyzed in Figs 4 and 5 about the obtained topologies, the two-stages method gives topologies with a wider coverage area than the 1-stage method. Therefore, the probability of the client being covered is higher, leading to increased NC. This is clearer from the results in Figs 8 and 9, where we plot NC as a function of the number of clients. Considering the case of using the MVO algorithm, the results are found as shown in Fig 8. We can easily observe that NC is inversely proportional to the number of clients for both methods. However, the two-stages method always outperforms the one-stage method in terms of NC. The average NC value increased by 4.03%. The results are also completely similar for the case of using the WOA algorithm, as shown in Fig 9, the average NC increases by an average of 3.98% if the two-stage method is used. The detailed data on the NC of the conventional method and the proposed method are clearly presented in Table 5, where we also summarize the simulation scenarios to determine the impact of the number of clients. It is evident that the suggested approach consistently yields a higher NC than the conventional method across all network instances with varying client counts.

**Table 5:** NC statistics versus total clients over 50 runs per scenario using the MVO algorithm with various approaches.

Algorithm	Router count	Average NC (%)		Max NC (%)		Min NC (%)		STD	
		one-stage	two-stages	one-stage	two-stages	one-stage	two-stages	one-stage	two-stages
MVO	100	87.41	92.10	92.00	96.80	75.20	86.40	2.90	2.49
	150	83.54	88.32	90.29	92.57	75.43	83.43	3.29	2.14
	200	84.03	87.63	89.78	91.56	78.67	81.33	2.39	2.29
	250	81.67	86.21	88.36	90.18	73.82	78.18	2.88	2.36
	300	83.48	86.36	89.23	92.31	77.23	80.31	2.72	2.35
	350	81.60	84.79	85.60	89.87	71.47	79.73	2.71	2.22
	400	81.98	85.38	87.29	89.41	75.53	80.94	2.61	2.31
WOA	100	79.02	82.04	87.00	88.00	73.00	73.00	3.22	3.00
	150	73.86	76.84	79.00	85.00	67.00	69.00	3.05	3.50
	200	72.40	75.12	77.00	83.00	65.00	69.00	2.72	3.46
	250	69.14	72.20	77.00	80.00	62.00	64.00	3.36	2.93
	300	70.68	73.94	79.00	81.00	65.00	66.00	2.98	3.20
	350	68.28	71.62	75.00	78.00	63.00	63.00	3.15	3.18
	400	69.22	72.40	76.00	79.00	64.00	67.00	2.79	3.38

**Fig 10:**
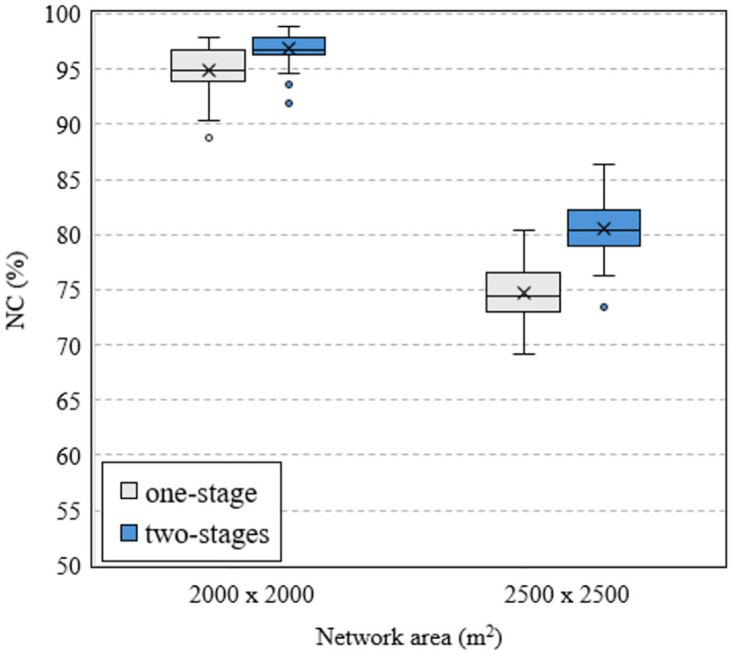
Compare NC versus the network area using MVO algorithm with different methods in case of 36 mesh routers.

**Fig 11:**
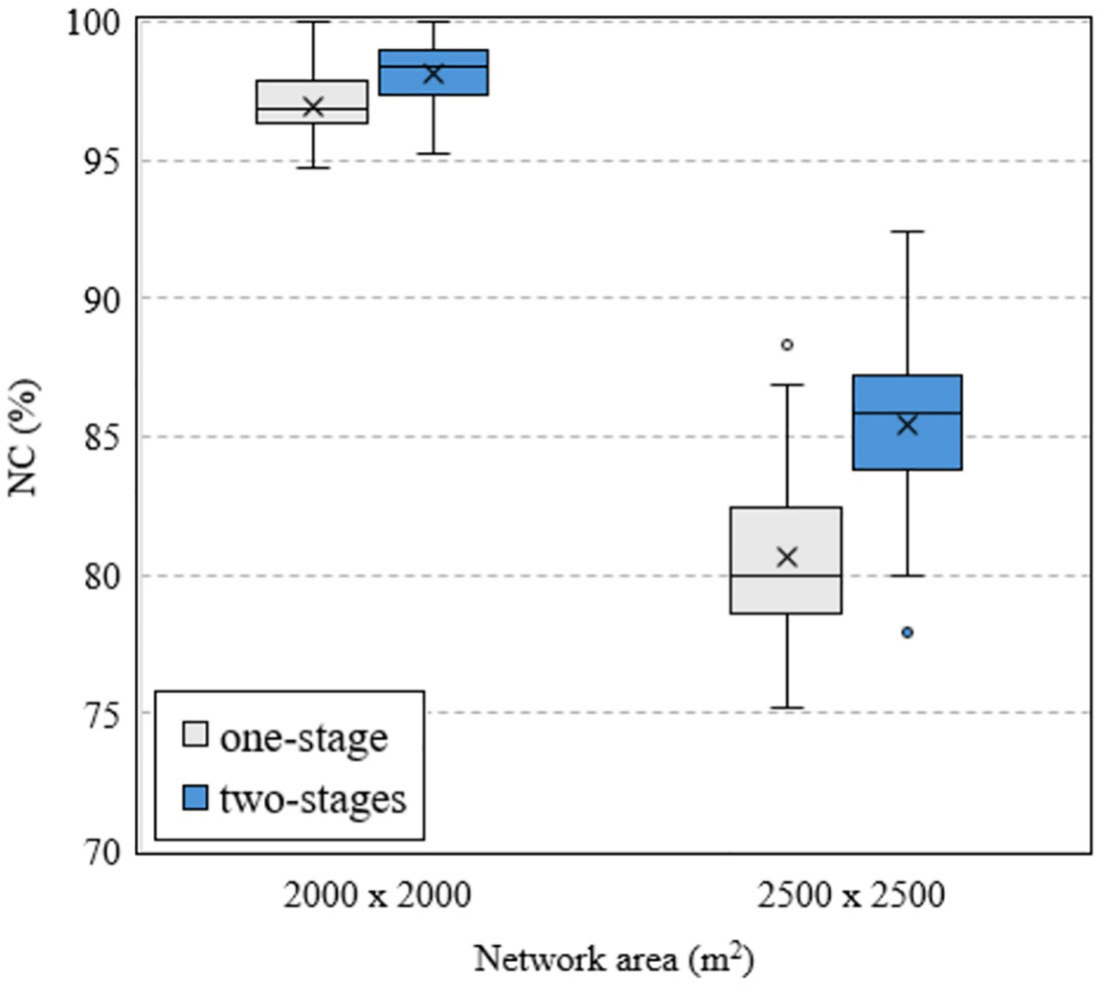
Compare NC versus the network area using MVO algorithm with different methods in case of 40 mesh routers.

In the case of a larger network area, the proposed method also provides higher efficiency than the traditional method. This is verified by the results shown in Figs 10 and 11, where we analyze the data of all 50 runs. We can observe that the larger the network area, the larger the difference in NC between the two methods. This implies that the proposed method provides higher efficiency in case of larger network area. Considering the case of using 36 routers, the box charts in Fig 10 show that when the network area is 2000 × 2000 [*m*^2^], the median values of the one-stage and two-stage methods are 94.89% and 96.77%, respectively, a difference of 1.88%. These values are 74.48% and 80.42%, a difference of 5.94% for the case of a network area of 2500 × 2500 [*m*^2^].

Through the results analyzed above, we conclude that the proposed method, two-stages, is highly effective in terms of research compared to the traditional method, one-stage. This is a very meaningful result, contributing to improving the performance of WMN.

## Conclusion

One of the methods to improve the WMN performance is to find the best solution to the RNP problem, that is, finding the best set of coordinates to place all mesh routers to achieve the given objectives. However, solving the RNP problem in WMN is difficult because it is NP-hard. As a result, this problem can only be solved using approximate optimization algorithms such as heuristics and meta-heuristics. This study proposed a new and effective method for solving the RNP problem. The idea behind this method is to solve the RNP problem in two stages using an optimal algorithm with fewer variables than the original RNP problem. In stage 1, we built an RNP sub problem using 15% to 20% of the number of routers, with the objective function of minimizing coverage overlap between routers to form a core network. Stage 2 is built into another RNP sub problem with the remaining number of routers, and the objective function is to maximize the network connectivity. Each of these sub-problems was solved using an approximate optimal algorithm. The experimental results demonstrate that our proposed method outperforms widely used NRP problem-solving methods in terms of network connectivity.

In future studies, we will further refine this approach by accounting for the additional quality of transmission limits and the mesh router load-balancing issue to enhance network performance.

## Supporting information

S1 Dataset (ZIP)
